# Abrasion Behaviour of Different Charcoal Toothpastes When Using Electric Toothbrushes

**DOI:** 10.3390/dj9080097

**Published:** 2021-08-20

**Authors:** Andreas Greuling, Johanna Maria Emke, Michael Eisenburger

**Affiliations:** Clinic for Prosthetic Dentistry and Biomedical Materials Science, Hannover Medical School, Carl-Neuberg-Straße 1, 30625 Hannover, Germany; Johanna.M.Emke@stud.mh-hannover.de (J.M.E.); Eisenburger.Michael@mh-hannover.de (M.E.)

**Keywords:** abrasion, brushing, charcoal, tooth wear, toothbrush

## Abstract

Objectives: The purpose of this in vitro study was to compare the abrasion behaviour of different charcoal toothpastes when brushing with electric toothbrushes on human enamel. Materials and Methods: A self-designed brushing machine was built using six commercially available electric toothbrushes in abrasion chambers. Each chamber was constantly supplied with a toothpaste–water mix. Pieces of human enamel, which were embedded in PMMA, were brushed for 4 h. Before and after brushing, profilometer measurements were performed in order to determine the substance loss due to brushing. Results: The following calculated mean removal values (mean ± SD) were found: (4.6 ± 0.6) µm (Group C: Splat Blackwood), (3.2 ± 0.9) µm (Group D: Curaprox Black is White), (2.3 ± 0.7) µm (Group B: Sensodyne Pro Schmelz), (1.7 ± 0.6) µm (Group A: Water), (1.4 ± 0.6) µm (Group E: Prokudent Black Brilliant). A post hoc Tukey HSD test (*p* = 0.05) showed that the results for Group A/B/E, Group B/D and Group C each lie within subsets that differ statistically significantly from the other subsets. Conclusions: Within the limitations of this in vitro study, it can be stated that some charcoal toothpastes lead to significantly higher abrasion on human enamel, when brushing with electric brushes. Clinical Relevance: As low-abrasion toothpaste is generally advisable, and some charcoal toothpastes should be viewed critically with regard to their abrasive properties.

## 1. Introduction

Currently, a lot of trends are caused by social media and influencers. One of those trends that has a rising popularity is the use of charcoal-containing tooth whitening pastes. These toothpastes are often described critically in the literature for several reasons [1,2,3,4,5]. Critique points are, for example, a lack of fluoride content [2,3], various reported scientifically unproven marketing claims and health risks [1,2] as well as possible unfavourable abrasion behaviour [4,5]. This manuscript focuses on the latter point.

When it comes to abrasion and erosion testing, there are different strategies used in the literature [6]. Some studies focus on the abrasive behaviour only [7,8,9,10], while other studies [11,12,13,14] perform erosion with acids followed up by abrasion cycles which can, depending, e.g., on the pH of the erosive liquid, have a significant impact. However, the use of erosive substances is strongly influenced by individual preferences and a test case without erosive substance is also of interest and allows for a meaningful comparison of different abrasive toothpastes. Furthermore, tests without an additional erosive cycle are more straightforward to perform and analyse when it comes to long brushing cycles which is why the experiments in this study are designed without erosive substances.

The abrasion behaviour of charcoal-based toothpastes has already been studied before using surface roughness analysis [4,5] but to the authors’ knowledge, human enamel substance loss has not been addressed quantitatively in the literature when using electric toothbrushes. In this work, we studied the substance loss associated with brushing for three charcoal-based toothpastes, a conventional toothpaste and water. The brushing was carried out in vitro using a self-built tooth-brushing machine, which is based on commercially available electric toothbrushes. The enamel substance loss was calculated via combining tactile profilometer measurements before and after brushing. 

## 2. Materials and Methods

**Figure 1:**
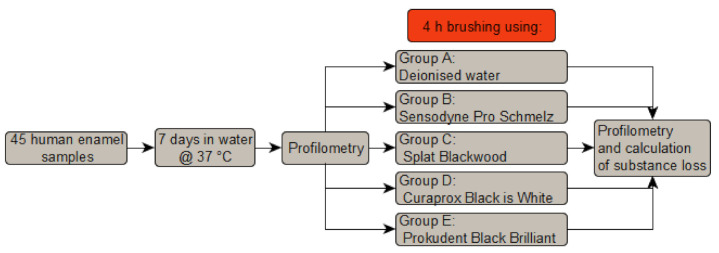
Study design.

In this work, the abrasion behaviour of human enamel by tooth brushing with different toothpastes was studied. In Group A, deionised water without any toothpaste was used. As a reference, conventional toothpaste with low abrasiveness (Sensodyne Pro Schmelz Repair Zahnschmelz, conventional (no charcoal)) was used in Group B. Furthermore, 3 different charcoal toothpastes were chosen (Group C: Splat Blackwood, Group D: Curaprox Black is White and Group E: Prokudent Black Brilliant). An overview of the study design can be found in Figure 1, details are described in this section. Information about the toothpastes is given in Table 1.

### 2.1. Toothbrushing Machine

**Figure 2:**
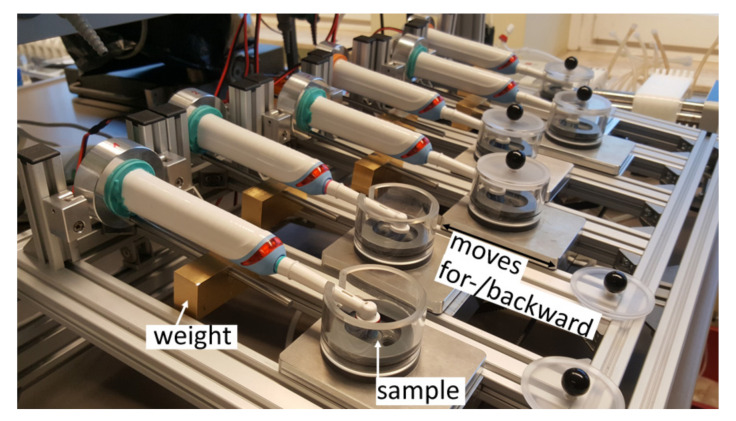
Custom-built automated tooth-brushing machine used in this study.

In this study, a self-designed brushing machine was used. It comprises six electric toothbrushes (Professional Care Pro 1000, Oral-B, Schwalbach am Taunus, Germany), in which the battery was replaced with a mains-connected power supply. The electric brushes are mounted on a metal frame (see Figure 2). The brushing heads are located in an abrasion chamber and the contact pressure can be adjusted with a weight. Each chamber has two holes which allow for a toothpaste/water mix to be pumped in and out via a peristaltic pump (IPC Ismatec, Cole-Parmer, Wertheim, Germany). The sample needs to be disc-shaped with a 12 mm radius and can be fixed in a notch between the inlet and outlet holes for the toothpaste slurry. When operating, the abrasion chambers move for- and backward a distance of 1 cm at 0.5 Hz to simulate the movement of an electric toothbrush in the mouth. This also distributes fresh toothpaste/water mix on the sample surface.

### 2.2. Sample Preparation

Altogether, 45 samples were prepared from human molars, which were free of caries. The teeth were gathered anonym at an external dental office, so patients’ ages or other information were not recorded. The teeth were cleaned and stored in a water-based solution with 70% ethanol. Before the start of sample preparation, the teeth were stored for 7 days in deionised water at 37 °C ± 1 K. After this, the teeth were quartered by cutting in the mesiodistal and bucco-lingual direction using a separating disc under water cooling. The root was separated and the pulpal side surface was flattened using a dental drill. At the end of this preparation step, 45 molar pieces were produced, in which the enamel side (later brushing side) was oriented in either the vestibular or oral direction. The enamel side was untreated and the back side flattened. The molar pieces were embedded in PMMA (Palapress, Kulzer), covering the whole piece and generating a flat disc. The enamel side of this disc was then polished using a polishing disc (DGD 70 µm, Buehler) until a small area of enamel was exposed. A part of this initially exposed area was later used for the measurement of substance loss. Care was taken that sufficient enamel remained so that the later brushing was on enamel only and did not penetrate into dentin. Small marks for later orientation were cut into the margin of the disc with a scalpel, followed by brief polishing of the enamel sample with silicon carbide (grain 1000). All samples were numbered and then randomly assigned to each of the five groups under the constraint that each group had no more than one piece of each tooth. The random assignment was performed using a Matlab script in order to avoid possible bias.

### 2.3. Profilometry and Calculation of Substance Loss

In order to calculate substance loss due to brushing, a surface scan was performed before and after brushing using a tactile profilometer (Dektak 150, Veeco, New York, NY, USA). A sample holder, which allowed for easy remounting in the same orientation, was used in this setup. As the scan only generates a surface map without absolute height information, a reference area was used to compare the two scans. During brushing, this reference area was covered with a thin PVC tape (tesafilm 4104, tesa SE, Norderstedt, Germany). The scan data (before and after brushing) were loaded into the software Gwyddion 2.34 [19]. The xyz data points had a spacing of 20 µm in the y direction and a 4 µm spacing in the x direction, with z as the height coordinate. In Gwyddion, a plane-level correction via 3 points on the reference area and a slight shift in the x/y direction were applied using a landmark (scalpel marks mentioned above) in order to correct for tilt and a small scan area mismatch. After this correction, the same area was selected in both data sets and the data were exported to a xyz file. A Matlab script was then used to calculate the mean height difference and to check for possible problems.

### 2.4. Brushing

All samples were brushed for 4 h under a load of 150 g (~1.5 N, based on ISO 11609:2017) using a new rotating brush head (Oral-B CrossAction, Procter&Gamble, Schwalbach am Taunus, Germany) for each sample. When assuming 10 s of brushing per day [12,20] in daily oral hygiene procedures on the same intraoral area, the simulated brushing time in the experiment refers to ~4 years of brushing. This is of course a rough estimate as the brushing time varies between individuals, meaning one should check this assumption when applying the conclusion for a certain clinical case. In the test groups (B–D), different toothpastes were used (see Figure 1). The toothpastes are: Sensodyne Pro Schmelz (GSK Consumer Healthcare, München, Germany), Splat Blackwood (SPLAT Germany, Berlin, Germany), Curaprox Black is White (curaden, Kriens, Switzerland), Prokudent Black Brilliant (Rossmann, Burgwedel, Germany). For all toothpastes, a ratio of 1 g toothpaste and 2 g deionised water was used, whereby the toothpaste–water mix was constantly mixed in the pumping reservoir. The 1:2 ratio has been reported in the literature as an “appropriate concentration for in vitro abrasion studies” [21] and is also recommended in ISO/TR 14569-1:2007. Additionally, one group was brushed using only water. 

### 2.5. Statistics

A univariate ANOVA including a Tukey HSD post hoc test was performed using SPSS 26 (IBM Deutschland GmbH, Ehningen, Germany) at a significance level of 0.05 to check the results for statistical differences.

## 3. Results

**Figure 3:**
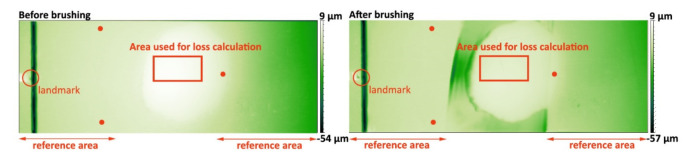
Profilometer height map of embedded enamel sample before and after brushing (exemplarily). The red dots indicate the points used for a plane-level correction. The cut marks in the reference area were used as landmarks for a slight x/y shift (see Section 2.3).

One of the resulting profilometer height maps is shown in Figure 3. In the left section of each image, one can see marks/pits that served as landmarks for x/y correction. The points chosen for the plane-level correction had a similar x/y position in the before/after scan. The height maps showed, after the brushing cycle, a small height loss of the exposed enamel. The PMMA embedding material around the enamel was far less resistant to brushing, which can be seen by the rather strong height decrease around the enamel. However, this is not the study’s focus as the enamel removal was only calculated in a region where enamel was exposed before brushing. In our study, the PMMA just acts as a sample holder.

**Figure 4:**
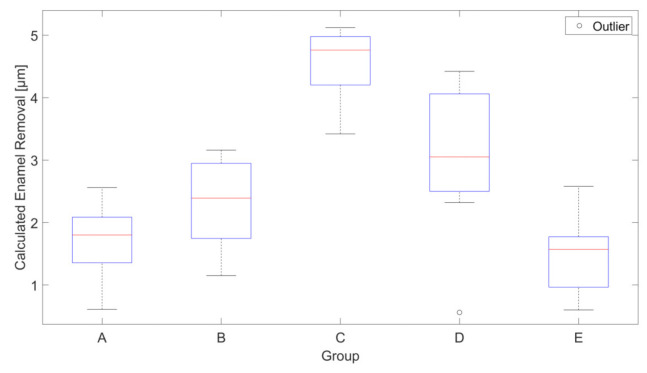
Calculated enamel removal after brushing with different toothpastes. N = 9 for all groups. Groups are A: water, B: Sensodyne Pro Schmelz, C: Splat Blackwood, D: Curaprox Black is White, E: Prokudent Black Brilliant.

Figure 4 shows the calculated enamel removal for all groups. In Group D, one sample showed possible preparation problems and was marked as an outlier for that reason. 

The calculated mean removal values are shown in Table 2. The results for the calculated removal show notable differences in the calculated mean enamel removal. A post hoc Tukey HSD test (*p* = 0.05) showed that the results for Group A/B/E, Group B/D and Group C each lie in a subset that differs statistically significantly from the other subsets. This result remains the same, regardless of whether the analysis is conducted with or without the outlier.

## 4. Discussion

When it comes to the question if charcoal toothpastes lead to higher abrasion with regard to the used reference, the answer of the study at hand is that some do, others do not. The highest mean enamel removal was (4.6 ± 0.6) µm in 4 h of electric brushing. When assuming 10 s brushing/day on a single area [12,20] and no further influences (erosion, saliva, remineralisation, etc.), this leads to about 93 µm enamel loss in 80 years. This estimation provides only an orientation as it is strongly dependent on the individual situation. Influences that lead to higher removal might be, for example, a longer brushing time, brushing with higher pressure, a different toothpaste/water mix or weakened enamel (e.g., by erosive substances, caries or genetic predisposition). Regarding the brushing time, one can also argue if the abrasion per time is similar for shorter brushing periods, which are closer to those seen in real oral hygiene. It should be checked in future work how the brushing duration influences the abrasion per time interval.

Comparing our abrasion results to the values for radioactive dentin abrasion (RDA) given in Table 1, Group B, C and D seem to lead to a higher enamel abrasion for higher RDA values. However, Group E, which has the highest RDA value according to the information from the distributor, leads to low abrasion in our tests. This may be due to different test procedures used for the RDA measurements. Unfortunately, details about the test laboratory and used methods are not available, so one should question the comparability of the different RDA values provided in Table 1. A lack of agreement between radiotracer and profilometry based methods was reported in the literature before [22]. Furthermore, the low abrasion of Group E might be caused by a lower concentration of charcoal or different particle size and structure caused by different manufacturing processes for the charcoal. The same is true for silica particles, which also cause abrasion and are part of all toothpastes tested in this study. It remains an open question how much of the abrasion can be attributed to the silica particles and how much to the charcoal particles. We plan to address this in future work in more detail.

Given the small number of toothpastes (one conventional, three charcoal toothpastes), one should also take care not to expand the results of this study to all available toothpastes, as the amount and type of particles responsible for abrasion can of course differ in other toothpastes. It should also be kept in mind that the conventional toothpaste, which was used as a reference, is a toothpaste with rather low abrasiveness.

Lima et al. [23] also investigated the substance loss for one of the toothpastes used in this study (Curaprox Black is White). They used a brushing simulator with manual toothbrushes and 30,000 brushing strokes and a force of 1.5 N. The authors obtained a substance loss of ~16 µm. Assuming a 1 Hz frequency, this would result in 8 h of brushing, resulting in 8 µm enamel loss in 4 h (it was 3.2 µm in 4 h in the study at hand). However, as the authors in [23] used bovine enamel and manual toothbrushes, differences are to be expected. 

As human molars were gathered anonym, the type of molars (impacted or not), the patients’ age and years in function are not known. Thus, differences in mineralisation can be expected, which can have an impact on the abrasion behaviour and can be seen as a limitation of the study. As the random assignment of the different tooth parts had the constraint that each group had no more than one piece of each tooth, this influence is somewhat reduced, but not completely removed.

When it comes to the method used in this study, the authors note that the two-sided covering was necessary for tilt correction. A one-sided cover—which was chosen in preliminary experiments—led to problems in the tilt correction and a higher standard error. The downside of the two-sided approach is, that the covering tape leads to a (thin) obstacle in the tooth-brushing path. However, the benefits of this approach outweigh the issues generated by a suboptimal tilt correction. Furthermore, a lower roughness in the reference area might also lead to improvements, whereas the influence in this study is probably not very high, as the authors did not use single points for the plane-level fit but point sets, which average some roughness issues out. As an alternative to the method used, approaches that measure the change in an indenter impression are also used in the literature [11,20,24]. The advantage is that there are no problems with tilt correction and good measurement accuracy. Joiner et al. report their measurement accuracy for a indent-based approach as 0.1 µm [24]. However, the indent changes the surface in a region where it matters. The abrasion might differ in the region of the indent, as the indent weakens the surface.

Erosion, which is not addressed in our experiments, can also play a significant role, as it can soften dentin and enamel, and lead to higher abrasion [12,25,26], whereas the abrasion in dentin is significantly higher than in enamel [12]. Attin et al. conclude that after contact with erosive substances, a remineralisation period before brushing should elapse in order to reduce wear [13]. The data of Eisenburger et al. suggest that a complete rehardening of softened enamel in vitro is reached after a remineralisation time of 6 h [27]. Furthermore, it is known that fluoride toothpastes can have a protective effect on eroded enamel subjected to brushing abrasion [28].

## 5. Conclusions

Given the data at hand, at first, it seems reasonably safe (referring to substance loss only) to use the charcoal toothpastes tested in this study, when only enamel is brushed for similar brushing times and pressures as tested without erosive or pre-damaged enamel. However, it is known that pre-damaged/softened enamel is more easily abraded [25]. If this becomes relevant, it might be a good idea to abstain from higher-abrasion toothpastes. However, it is generally advisable to use a low-abrasion toothpaste. In addition, further work is needed for a more complete view that allows for a more accurate quantification of substance loss, whitening effect and plaque removal. Without this additional information, clinical applications are limited.

## Figures and Tables

**Table 1:** Information about the ingredients and relative dentin abrasion (RDA) value of the used toothpastes.

Group	Commercial Brand	Ingredients	RDA
A	-	Aqua	-
B	Sensodyne Pro Schmelz Repair Zahnschmelz, conventional (no charcoal)	Hydrated Silica, Aqua, Sorbitol, Glycerin, Potassium Nitrate, PEG-6, Sodium Lactate, Cocamidopropyl Betaine, Aroma, Titanium Dioxide, Xanthan Gum, Sodium Saccharin, Sodium Fluoride (1450 ppm F^−^), PVM/MA Copolymer, Sodium Hydroxide, Limonene	35 (±15%) [15]
C	Splat Blackwood	Hydrated Silica, Charcoal Powder, Aqua, Hydrogenated Starch Hydrolysate, Glycerin, Maltooligosyl Glucoside, Sodium Lauroyl Sarcosinate, Cellulose Gum, Aroma, Capryloyl/Caproyl Methyl Glucamide, Lauroyl/Myristoyl Methyl Glucamide, Sodium Benzoate, Stevia Rebaudiana Leaf Extract, Potassium Sorbate, Menthol o-Cymen-5-ol, Juniperus Communis Sprout Extract, Limonene	75 [16]
D	Curaprox Black is White	Hydrated Silica, Charcoal Powder, Aqua, Sorbitol, Glycerin, Aroma, Decyl Glucoside, Cocamidropropyl Betaine, Sodium Monofluorophosphate 950 ppm F^-^, Tocopherol, Xanthan Gum, Maltodextrin, Mica, Hydroxylapatite (Nano), Potassium Acesulfame, Titanium Dioxide, Microcrystalline Cellulose, Sodium Chloride, Citrus Limon Peel Oil, Sodium Hydroxide, Zea Mays Starch, Amyloglucosidase, Glucose Oxidase, Urtica Dioca Leaf Extract, Potassium Thiocyanate, Cetearyl Alcohol, Hydrogenated Lecithin, Menthyl Lactate, Methyl Diisopropyl Propionamide, Ethyl Menthane Carboxamide, Stearic Acid, Mannitol, Sodium Bisulfite, Tin Oxide, Lactoperoxidase, Limonene	50 [17]
E	Prokudent Black Brilliant	Hydrated Silica, Charcoal Powder, Aqua, Sorbitol, Propylene Glycol, Pentasodium Triposphate, Tetrapotassium Pyrophosphate, Sodium C14-16 Olefin Sulfonate, Aroma, Disodium Pyrophosphate, Xanthan Gum, Menthol, Sodium Fluoride (1450 ppm F^−^), Sodium Saccharin	120 [18]

**Table 2:** Calculated enamel removal after brushing with different toothpastes.

Group	Commercial Brand	Mean Removal [µm]	Standard Deviation [µm]
A	Water	1.7	0.6
B	Sensodyne Pro Schmelz Repair Zahnschmelz, conventional (no charcoal)	2.3	0.7
C	Splat Blackwood	4.6	0.6
D	Curaprox Black is White	3.2	0.9
E	Prokudent Black Brilliant	1.4	0.6

## Data Availability

The data are available on reasonable request.
